# A novel vehicle-like drug delivery 3D printing scaffold and its applications for a rat femoral bone repairing *in vitro* and *in vivo*

**DOI:** 10.7150/ijbs.37552

**Published:** 2020-04-01

**Authors:** Hui Wang, Zhengwei Deng, Jing Chen, Xin Qi, Libing Pang, Bocai Lin, Yan Teik Yuin Adib, Na Miao, Deping Wang, Yadong Zhang, Jiusheng Li, Xiangqiong Zeng

**Affiliations:** 1 Laboratory for Advance Lubricating Materials, Shanghai Advanced Research Institute, Chinese Academy of Sciences, Shanghai 201210, China.; 2 Department of Orthopedics, Fengxian District Central Hospital Affiliated of Shanghai University of Medicine&Health Sciences, 279 zhouzhu road, Shanghai 220120, People's Republic of China.; 3 School of Materials Science and Engineering, Tongji University, Shanghai 201804, China.; 4 School of Life Science & Chemical Technology, Ngee Ann Polytechnic, Singapore 599489; 5 Graduate School, Shanghai University of Traditional Chinese Medicine, Shanghai 201203, China; 6 Department of Pediatrics, Maternal and Child Health Hospital of Zaozhuang City, Shandong, China.

**Keywords:** vehicle-like 3D printing scaffold, control release, mesoporous bioactive glass, 1393 bioactive glass, bone treatment

## Abstract

The high surface area ratio and special structure of mesoporous bioactive glass (MBG) endow it with excellent physical adsorption of various drugs without destroying the chemical activity. Silicate 1393 bioactive glass (1393) is famous for its fantastic biodegradability and osteogenesis. Herein, we have built a novel vehicle-like drug delivery 3D printing scaffold with multiplexed drug delivery capacity by coating MBG on the surface of 1393 (1393@MBG). Furthermore, we have applied DEX and BMP-2 on the 1393@MBG scaffold to endow it with antibacterial and osteogenic properties. Results indicated that this 1393@MBG scaffold could effectively load and controlled release BMP-2, DNA and DEX, which can be applied for orthopedic treatment. The *in vitro* study showed that the DEX loaded 1393@MBG exhibited excellent antibacterial ability, which was evaluated by *Staphylococcus aureus* (*S. aureus*), and the BMP-2 loaded 1393@MBG can improve the alkaline phosphatase (ALP) activity and upregulate the expression of osteogenesis-related genes (OCN and RUNX2) of human bone mesenchymal stem cells (hBMSCs). Moreover, the *in vivo* study further confirmed that the BMP-2 loaded 1393@MBG group showed better osteogenic capacity as compared to that of the 1393 group in a rat femoral defect. Together, these results suggested that the vehicle-like drug delivery 3D printing scaffold 1393@MBG could be a promising candidate for bone repair and relative bone disease treatment.

## Introduction

There is an urgent demand for bone regeneration biomaterials because of the increasing frequency of traffic accidents, industrial contingencies, and natural disasters. Although efforts have been devoted to develop different kinds of tissue implants, the repair of bone defects still faces many challenges in current regenerative medical research 1, 2, including the differing degradation rates between implants and new tissues. Another challenge is reconciling the bone regeneration and treatment during the repair of bone defects caused by diseases, such as bone tumors or osteoporosis 3. Clinically, some chemical molecular drugs or cytokines, such as bone morphogenetic protein 2 (BMP-2), have been employed to treat bone defects. The activity of these sensitive drugs or cytokines should not be neglected. The growth factor BMP-2 is well known as a morphogen to induce osteogenesis from stem cells 49, it is also one of common cytokines that usually be used to enhance bone regeneration. Dexamethasone (DEX) is a synthetic corticosteroid chemical drug that can be used to treat a variety of symptoms, including rheumatic diseases. Furthermore, some tissue or cellular micro-environment changes may cause orthopedic diseases, which require clinical molecular probes for detection, such as antibody or DNA probes. In the view of these situations, there is an urgent need to develop the bone repairing grafts with high feasibility of multi-functionalization or vehicle-like capacity for good drug delivery 4.

The development of synthetic scaffolds for application in bone tissue regeneration and disease treatment, should possess combined optimum properties such as pore architecture, mechanical strength, bioactivity, degradation and controllable drug-delivery ability; the development of new 3D porous scaffolds for bone regeneration has therefore become the focus of many recent studies 5, 6. Bioactive glass shares some properties with other bioactive inorganic materials such as hydroxylapatite (HA) and beta-tricalcium phosphate (β-TCP), in that it can bond with host bone tissue, however bioglass has better bioactivity and degradation properties 3, 12, 13. Bioactive glass soaked in simulated body fluids (SBF) can degrade and convert to HA, forming a firm bond to bone tissue, and in the meanwhile, bioactive glass can release functional ions, such as silicon ions and calcium ions, which promote bone regeneration 7, 8. Among which, bioactive silicate glass (1393) has been selected because it can match the rate of new bone growth due to its higher reactivity, degradation and conversion to hydroxyapatite 9-11. Simultaneously, mesoporous bioactive glass (MBG) has a highly ordered mesopore channel structure with pore sizes ranging from 5 to 20 nm. An important feature of MBG is that it has a greatly improved surface area and pore volume, which shows superior bioactivity compared to non-mesoporous bioglass 16, 47, 48.

Recently, 3D printing technology has been widely used to fabricate bioglass and bioceramic scaffolds with high porosity, pore connectivity and mechanical strength 14-17. With the use of 3D printing technology, bone tissue scaffolds can be designed and customized 18. Therefore, 3D printing technology has obvious advantages in the preparation of bone tissue scaffolds. The 3D printing bioactive glass scaffold is closer to an ideal bone tissue graft. It has high porosity, appropriate mechanical strength, excellent biocompatibility and bioactivity, can degrade and convert to hydroxyl apatite comparatively quicker, and can promote stem cells proliferation and differentiation. However, it is not an ideal bone tissue graft as it requires additional versatility including the capacity for drug delivery. Herein, applying a mesoporous bioactive glass coating onto the surface is a viable option for the drug delivering capacity of bioactive glass scaffolds 19. With mesopores on the surface, DNA, cytokines and some drugs can be loaded onto the scaffold, meeting the requirements of disease treatment or further enhanced bone regeneration 19-22.

The focus of this study was to integrate MBG with 3D printing basic 1393 bioactive glass scaffolds (1393@MBG). Furthermore, we have applied DEX and BMP-2 on the scaffold to endow it with antibacterial and osteogenic properties. The mechanism of drug load and controlled release ability was detailly investigated, and the bioactivity and osteogenesis changes of the BMP-2 loaded 1393@MBG scaffolds were evaluated by culturing with hBMSCs *in vitro* and a rat femoral defect model *in vivo*. The antibacterial ability of DEX loaded 1393@MBG was quantitatively evaluated by *Staphylococcus aureus* (*S. aureus*). It is expected that the findings in this research will shine a new light on the clinical orthopedics and tissue engineering field.

## Experimental Procedure

Materials: The composition of basic 1393 bioactive glass goes as following (6Na_2_O, 8K_2_O, 8MgO, 22CaO, 54SiO_2_, 2P_2_O_5_; mol%). The corresponding metal oxide carbonate and sulfate of the above material were all purchased from Sinopharm Chemical Reagent Co., Ltd. (China). Tetraethyl orthosilicate (TEOS), Ca(NO_3_)_2_•4H_2_O, triethyl phosphate, and EO20PO70EO20 (P123) for the synthesis of MBG were purchased from Sigma-Aldrich Chemical Co., Ltd.. Other reagents were purchased from Sinopharm Chemical Reagent Co., Ltd. (China). All reagents (analytical grade) were used as received without further purification.

### Preparation of 1393@MBG scaffolds

#### Preparation of MBG so-gel

The MBG so-gel solution was synthesized by using EO20PO70EO20 (P123), a type of nonionic block copolymer, as a pore-forming templating agent as reported previously 20. Firstly, P123 (4.0 g), TEOS (6.7 g), Ca(NO_3_)_2_•4H_2_O (1.4 g) and TEP (0.73 g) were dissolved in ethanol (60 g), after that 0.5 M HCl (1.0 g) was added and then the solution was stirred vigorously for 2 days at room temperature (RT).

#### Preparation of basic scaffolds by 3D printing technology

The basic 1393 bioactive glass (designated as 1393) scaffolds were created using 3D printing technology. The corresponding metal oxide carbonate and sulfate were mixed, and then heated at 1200 ºC for 1 hour to form the glass. Next, the glass was crushed and grounded, and then sieved to small particles with average size around 30 µm.

The 4^th^ 3D printing Bioplotter™ (EnvisionTEC GmbH, Germany) was applied in this study for fabricating the 3D basic glass scaffolds. Firstly, mixed the BG glass powder, ethyl cellulose and ethanol (3 : 0.12 : 2.5, wt%) together to make the 3D printing glass slurry. The prepared slurry was then introduced into a polyethylene syringe and then fixed onto the printing machine. Subsequently, load the cylindrical models into the 3D printing machine and plot the designed scaffold layer-by-layer.

Dry the printed scaffold in air at RT for 24 hours. Heat the scaffold at 300 ℃ for 4 hours to burn out the polymer and then at 580 ℃ for 2 hours to sinner the parent BG scaffold to a dense 3D scaffold. Lastly, use the cutting machine to modify the scaffold into uniform cylindrical (5 mm in diameter × 2 mm).

#### Preparation of 1393@MBG scaffolds

 The suspension coating method was applied to prepare the 1393@MBG scaffolds. The basic 1393 scaffold was introduced into a centrifuge tube and the above obtained MBG so-gel solution was dropped on the scaffolds. The spinner machine will coat the scaffold using centrifugal force at a speed of 6000 rpm and then dried in the air. The coating and drying procedure was repeated for 10 times and the scaffold was then removed from the solution. The scaffold obtained above was dried for 24 hours under vacuum at 60 ℃ achieving the embedment of MBG onto the surface 1393 scaffold.

### Characterization of the fabricated scaffolds

The morphology of the above obtained scaffolds was characterized by field emission scanning electron microscopy (FESEM, Hitachi S-4700; Tokyo, Japan). In order to determine the thickness of the coating layer, the scaffolds were clotted in polymethylmethacrylate (PMMA) and were cut and coated with gold and monitored by FESEM. X-ray diffraction (XRD, Rigaku, Tokyo, Japan) was used to analyze the glass powder and the presence of any crystalline phases. Transmission electron microscopy (TEM, JEM-2100F, JEOL) was carried out to look at the structure of the nano-channels within the MBG glass. Surface area measurements were carried out by nitrogen gas adsorption in a micromeritics Gemini VII 2390 gas adsorption analyzer (Micromeritics, Georgia, USA). Surface area was then calculated using the Brunauer Emmett Teller (BET) theory, and pore size distribution and pore volume were estimated using the Barrett-Joyner-Halenda (BJH) schema. A mechanical testing machine (CMY6104 SANS) at a crosshead speed of 0.5 mm/min and a 1 kN load cell was used to test the compressive strength of the cylindrical scaffolds.

### The bioactivity, degradation, mineralization of scaffolds *in vitro*

The bioactivity properties, degradation rate and mineralization of the above as-fabricated scaffolds were evaluated by being immersed in simulated body fluid (SBF) 23. The ratio of 0.1 g to 10 ml of as-fabricated scaffolds immersed in SBF solution was applied to evaluate the sample degradation process by monitoring the pH value change of the 1393, 1393@MBG sample and MBG glass powder immersed in SBF 24. The concentration of dissolved ions released from 1393 and 1393@MBG scaffolds in SBF were tested by inductively coupled plasma atomic emission spectroscopy (ICP-AES, Varian, USA). The average surface roughness (Ra) and morphology change of the scaffolds after immersion in SBF was measured by non-contact 3D surface interferometer (Bruker, contour GT, USA).

### Evaluation the drug loading and controlled release ability of 1393@MBG scaffolds *in vitro*

As a proof-of-concept experiment, a designed ssDNA (5'-FAM-CCCTAACCCTAACCCTAACCCT-3') was chosen as a model to test the feasibility of the after said DNA probe. Bone morphogenetic protein 2 (BMP-2, 12.5kDa) and dexamethasone (DEX, C_22_H_29_FO_5_, 392), that are commonly applied as clinical therapy drugs for bone repair, were chosen to evaluate the controlled release ability and delivery mechanism. For the drug loading process, DNA, DEX and BMP-2 were dissolved in SBF to 0.1 mg/ml, then added to 1.0 g of scaffolds mixed with 50 ml of drug solution and stirred for 24 hours. After that, the DNA, DEX and BMP-2 loaded scaffolds were collected and dried for 1 hour at 37 °C. The drug release ability of the scaffolds was evaluated by immersing the scaffolds in SBF with a ratio of 1.0 g to100 ml. The estimation of the released drug concentration was determined by testing the absorbance values at 241 nm for DEX, 260 nm for DNA and 567 nm for BMP-2 after staining with ninhydrin 25. The rates of loading and release were determined by NanoDrop 2000C Spectrophotometer.

### *In vitro* antibacterial activity of the scaffolds

*S.aureus* (ATCC 25923) was used as the *in vitro* bacteria model to evaluate the antibacterial activity of the above prepared DEX loaded scaffolds. For the quantitative analysis of the antibacterial activity of the prepared DEX loaded scaffolds, *S. aureus* bacterial suspension was added to 5 mL LB liquid medium with an OD value between 0.1 and 0.2 at 600 nm in individual test tubes. Then, 0.45 g scaffolds were added to each test tube and incubated for 24 hours at 37℃. The absorbance of the bacterial suspension was measured at 600 nm using a Lambda 25 UV-Vis spectrophotometer 45.

### *In vitro* cell responses of the scaffold

As-fabricated BMP-2 loaded scaffolds were sterilized at ultraviolet light for 2 hours before use. After that, the scaffolds were incubated in DMEM supplemented with 10% FCS at 37 ℃ in a humidified atmosphere of 5% CO_2_ with seeded hBMSCs. For the morphology observation, the scaffolds with hBMSCs attached were rinsed with PBS, post-fixed in 1% osmium tetroxide in PBS, dehydrated with a graded series of ethanol and then air dried. Finally, these scaffolds were covered with gold and observed by FESEM at the accelerating voltage of 15kV.

For calcein AM/PI staining, all the cells were stained by co-staining dye solution (200 μL) containing calcein-AM (10 mM) and PI (10 mM) for 15 minutes at 37 °C, in the dark. Then, the excess staining solution was removed, and the cells were carefully washed with PBS twice. Finally, PBS (200 μL) was added, and the cells were subsequently visualized under a confocal laser scanning microscope (CLSM, FV1000, Olympus). Living cells and dead cells were stained by calcein AM to exhibit green fluorescence (λ_ex_ = 490 nm, λ_em_ = 515 nm) and PI to exhibit red fluorescence (λ_ex_ = 528 nm, λ_em_ = 617 nm), respectively.

The cytotoxicity and proliferation of the hBMSCs cells on the scaffolds were evaluated using CCK-8 to test the absorbance at 450 nm. The hBMSCs (1×10^4^) were cultured on the scaffolds following the procedure for 1, 3 and 7 days. After culturing, the attached hBMSCs were rinsed off with PBS. After which, 40 ml of the CCK-8 solution and 360 ml of the culture medium were added for 4 hours at each time point, and the absorbance was measured at 450 nm using a micro-plate reader (Bio-Rad 680, USA).

After 10 days of osteogenic differentiation, the osteoblast phenotype of hBMSCs was evaluated via ALP staining. ALP staining was performed using an ALP kit (Sigma-Aldrich) following the manufacturer's instructions. The ALP-positive cells were stained a purple color. In addition, Alizarin Red S staining (ARS, Millipore, Billerica, MA) was performed to examine the mineralization of the hBMSCs. ARS stained the calcium-rich deposits secreted by cells a red color. The cells cultured in osteogenic medium for 10 days were washed with PBS twice, fixed with 10% formaldehyde, and stained with ARS.

The differentiation of the hBMSCs on each scaffold was measured by the alkaline phosphatase (ALP) activity. 1×10^5^ cells (n = 6) were seeded in the scaffolds for 7 and 14 days. After that these scaffolds were rinsed with PBS and 50 mM Tris buffer, and then lysed in 200 ml 0.2% Triton X-100. Lysates were sonicated after being centrifuged. Finally, 50 ml supernatant was mixed with 150 ml working solution according to the manufacturer`s protocol (Beyotime; China). The results were measured at 405 nm by a plate reader (Bio-Rad 680, USA).

The representative osteogenesis related gene expression including RUNX2 and OCN in hBMSCs were also investigated. The primer sequences used in this study were provided by BioSune Biotechnology Co., Ltd. (Shanghai, China). To calculate the relative value of gene expression, the value of CTR groups was set as 1 and the GAPDH was used as a reference gene.

### *In vivo* evaluation of the new bone formation

The animal treatment procedures which were according to the guidelines were approved by the Shanghai Jiao Tong University Ethics Committee. Three months old SD rats (250-300 g) were used to fabricate rat femoral defects models. The Femoral cylindrical defects were created, which were standardized at 3 mm in diameter, and internally penetrated approximately 3 mm deep according to the reference 46. After 3 months post operation, the rats were sacrificed, and the histological specimens were harvested for following Micro-CT and histologic analysis.

A Micro-CT scanner (Skyscan 1176, Kontich, Belgium) was used to reconstruct images by software. The relative bone volume fraction (bone volume/tissue volume, BV/TV) and the bone mineral density (BMD) were analyzed using the software. Van Gieson's picrofuchsin staining was applied to analyze the new bone tissue formation. The histological staining observation image was examined by Image Pro Plus^TM^ (Media Cybernetics, Silver Springs, MD).

### Statistical analysis

All data were expressed as means ± standard deviation (SD) and were analyzed using one-way ANOVA for independent sampling. The criteria for statistical significance were *p < 0.05

## Results

### The physical properties of the as-fabricated scaffolds

**Figure 1:**
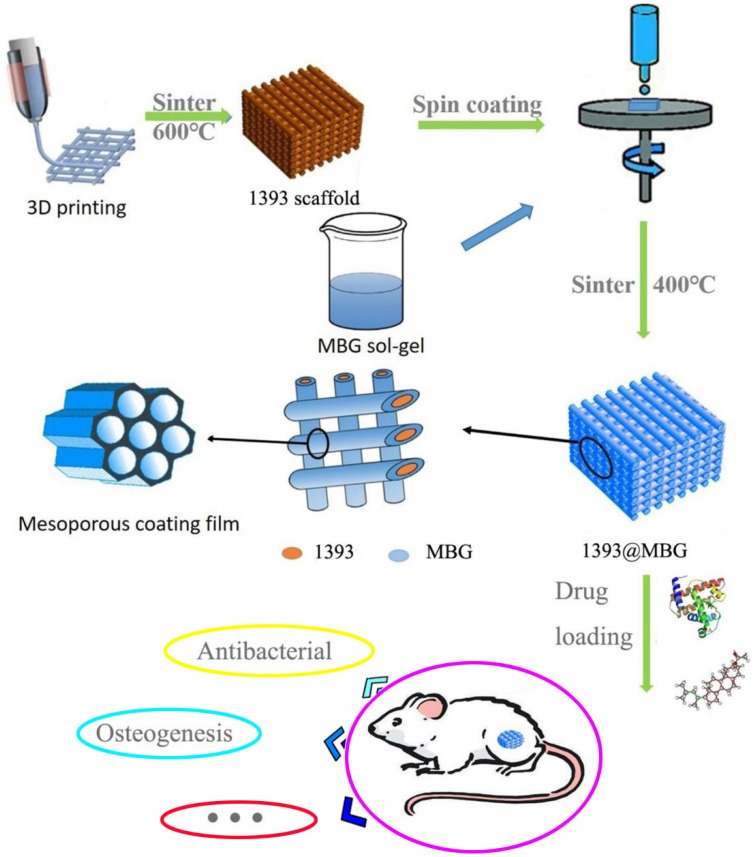
The flow chart of fabricating a novel drug delivery 1393@MBG scaffold built by 3D printing technology for bone repairing.

**Figure 2:**
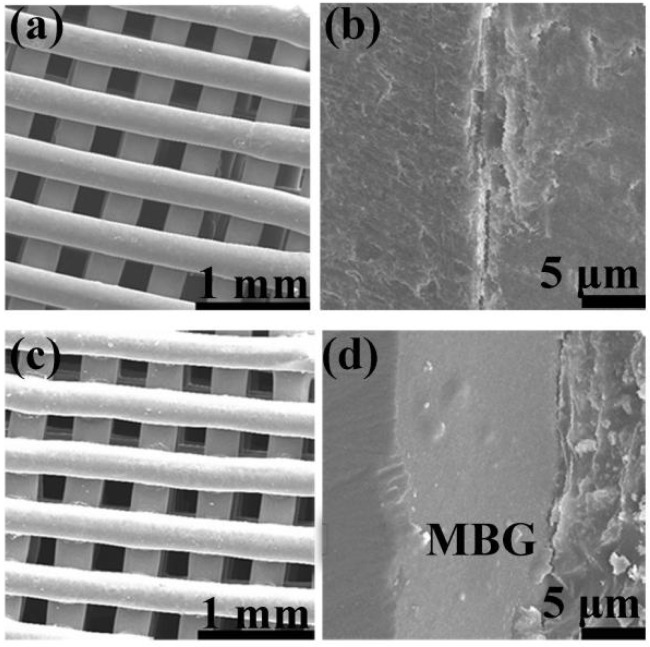
FESEM images of (a, c) as fabricated 1393 and 1393@MBG scaffold; (b, d) the cross section of as fabricated 1393 and 1393@MBG scaffold.

**Figure 3:**
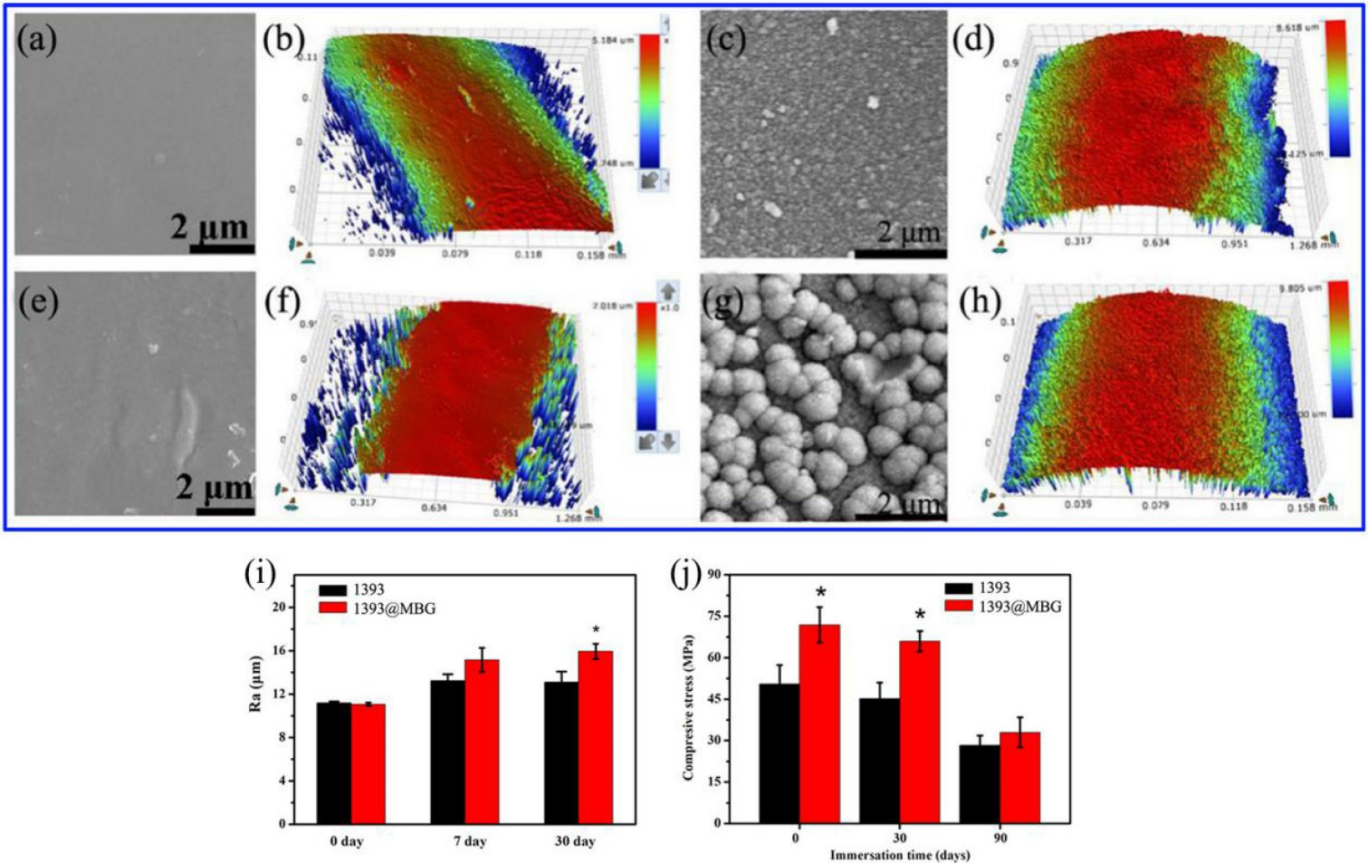
(a, b) FESEM images and the surface profile of as fabricated 1393 scaffold; (c, d) after immersed scaffold; (e, f) FESEM image and the surface profile of as fabricated 1393@MBG scaffold; (g, h) after immersed scaffold; (i) The Ra of 1393 and 1393@MBG scaffold surface when immersed from 0 to 90 days; (j) The compressive strength of the 1393 and 1393@MBG scaffold on day 0, 30 and 90. mean ± SD, n = 5. *Significant difference when compared to 1393 (*p* < 0.05).

The basic 1393 scaffold has been prepared successfully by 3D printing technique (Fig. 1). FESEM image indicated that the as fabricated 1393 scaffold had quite uniformed macroporous structures (Fig. 2), and the pore size was around 300 μm. The morphology of the as fabricated scaffold was consistent with the previously set parameters in the software. No distinct differences can be observed both for the microstructure and the surface roughness of 1393 and 1393@MBG scaffolds in the beginning as can be seen in Fig. 2. At the 90^th^ day, images of FESEM and 3D profile test were obtained and there were prominent differences between the 1393 and 1393@MBG scaffolds. Some parts on the surface of the 1393@MBG group were peeled off and it was populated with ball like structures. The corresponding Ra data demonstrated that there was a significant difference between the 1393@MBG and 1393 group (Fig. 3 i). The 1393@MBG scaffold exhibited a higher Ra due to the high activity of MBG. The mesoporous diameter of MBG was nearing 5 nm, which endowed the materials with higher surface area. This property provides more nucleation sites and the material exhibits stronger absorption behavior. Both these two points were beneficial for HA generation, while the 1393 group only possessed a bare surface.

The compressive strengths of the 1393 scaffold on day 0, 30 and 90 were 50.43 ± 6.80, 45.15 ± 5.82 and 28.26 ± 3.57 MPa, respectively, while the corresponding compressive strengths of 1393@MBG scaffold were 71.86 ± 6.40, 65.85 ± 3.79 and 32.94 ± 5.43 MPa, respectively (Fig. 3 j). This data clearly demonstrated that the MBG coating can improve the compressive strength of the basic scaffold. The MBG coating can physically increase the compressive strength of the scaffold and reduce the speed of biodegradation, which can be distinctly observed from the data of day 30. However, finally on day 90, the compressive strengths of 1393@MBG and 1393 scaffolds were 32.94 MPa and 28.26MPa, respectively. This is due to the degradation of MBG coating film, thus leaving only the basic 1393 scaffold, which resulted in there being similar compressive strengths between the 1393@MBG and 1393 scaffolds. The compressive strength of the scaffolds meet the requirement of the human trabecular bone (2-12 MPa) 26.

The XRD data indicated that the generation of HA by MBG possesses higher chemical ability, which is faster than that of 1393 as shown in Fig. S1. A typical amorphous glass can be clearly observed in both 1393 and MBG glass powder from the XRD analysis before immersion, all these samples showed a broad reflection at 30°. When we looked into the results, the data showed that the diffraction patterns of the fabricated MBG had a low intensity and SiO_2_ peak, as immersion time increased to either 30 days or 90 days, a clear and sharp HA diffraction (JCPDS 72-1243) peak can be observed in the pattern. However, small peaks were observed for the 1393 group when the immersion time was 90 day.

In Fig. S2, the pH value change of the immersion solution and released ions showed similar trends. The change in pH value of MBG sample was rapid during the first 3-7 days which then slowed down after 10 days. Similarly, the rate of Si ions release was rapid in the first 10-12 days and then decreased to a slower rate. The pH values between pure MBG, 1393@MBG and 1393 were similar. For the Si ions release, the rate at which ions were released from 1393@MBG coating is much slower than that from 1393. The pure MBG powder and 1393@MBG scaffolds showed higher Si ions release rates than that of 1393, which indicated the higher active chemical capability of MBG. MBG degraded faster than 1393 due to its superior bioactivity and biodegradability. For the MBG coating on the 1393@MBG sample, the coating film could clearly retard the degradation.

### Evaluation of the drug controlled release ability of 1393@MBG scaffold

**Figure 4:**
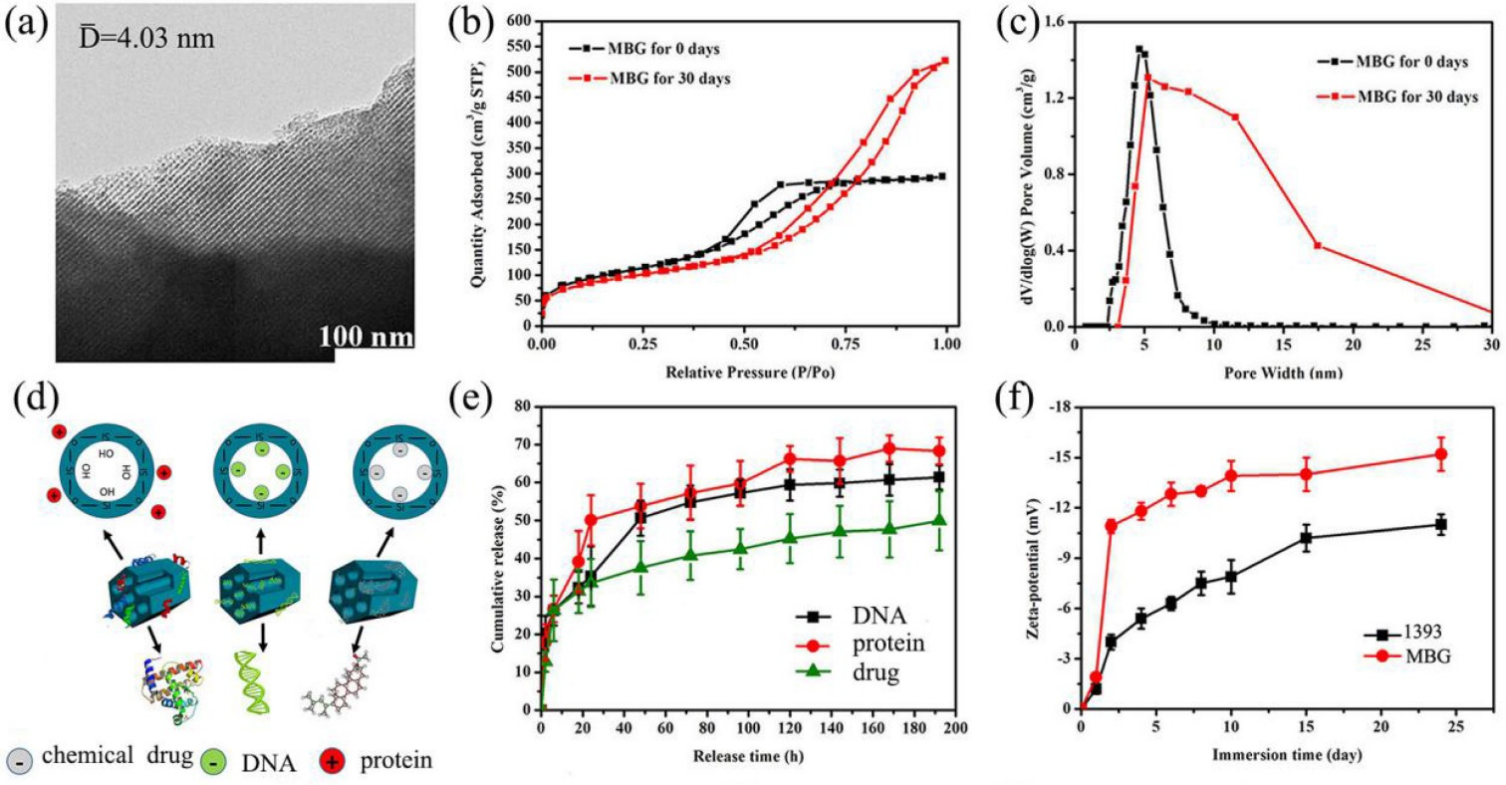
(a) TEM image of the as made MBG powder; (b) N_2_ adsorption-desorption isotherms and (c) the corresponding pore size distributions of MBG powders; (d) illustrator of the mechanism of MBG loading protein, DNA and chemical drug; (e) chemical drug, DNA and protein release profiles from the MBG powders in SBF at 37 ℃; (f) Zeta-potential of 1393 and MBG glass powder immersed in SBF at 37 °C as a function of immersion time; mean ± SD, n = 5.

TEM image (Fig. 4 a) clearly exhibited that the as made MBG glass powder possesses well-ordered mesoporous channels, which was similar to the previous result 28. BET results showed that the mesoporous structure of MBG could be kept for at least 30 days in SBF, this will endow basic materials with good drug loading and release ability. The surface area of the MBG powder was 371.66 m^2^/g which was shown by the N_2_ adsorption test. The total volume at single point adsorption was P/P0 = 0.65, which indicated that the main pores were mesopores and the average mesopore size was 4.03 nm. When the MBG sample was immersed in SBF for 30 days, the BET data showed that the MBG has accumulated pores which formed by generated HA. The pores size distribution changed, and surface area also increased to 407 m^2^/g.

Obviously, Zeta-potential of 1393 and MBG samples increased as the immersion time went on in Fig. 4 f. We can also easily see that the Zeta potential showed increased negative charge as time went on, which indicated that increasing amounts of OH groups were absorbed and HA was generated on the surface of MBG and 1393 29.

In this study, we focused on the drug release ability by loading DNA, DEX and BMP-2 on scaffolds. From the data, MBG can load and control the release of drugs by the physical absorption without affecting the structure and chemical properties of the drugs. Referring to Fig. 4 d, BMP-2 has the fastest release rate followed by DNA then DEX which might be attributed to the size of the molecules. The DEX was totally free shuttle in the pore due to it being much smaller than the MBG pores. The DNA was small enough to enter the pore while the size of BMP-2 (12.5 kDa) was too large to enter the mesoporous channels. Also, the scaffolds will be easier to release the larger molecule. At the same time, the functional groups of these samples also have an effect on the drug release rate 30. The main functional groups of DEX, DNA and BMP-2 were cholesteryl, phosphate and amino group, respectively, which will exhibit a negative, negative and positive charge in a water solution, respectively. The main group of MBG was silicon oxide group, thus when immersed in a water solution the surface of MBG will exhibit a positive charge which will more easily absorb negative materials such as -OH groups, DEX and DNA. So, this may explain the phenomenon that BMP-2 release was better than that of DNA and DEX, in the order, BMP-2 > DNA > DEX.

### Antibacterial activity of scaffolds *in vitro*

The DEX loaded 1393@MBG scaffold can better inhibit the proliferation of *S. aureus*. than that of the DEX loaded basic 1393 scaffold due to the higher amount of drug loaded. These observations were supported by the quantitative analysis of antibacterial activity of the scaffolds in LB liquid medium. The 1393 group showed a bacterial inhibition of 20.3% while the bacterial inhibition increased to 70.5% for that of 1393@MBG group.

### Cell responses of the scaffolds *in vitro*

**Figure 6:**
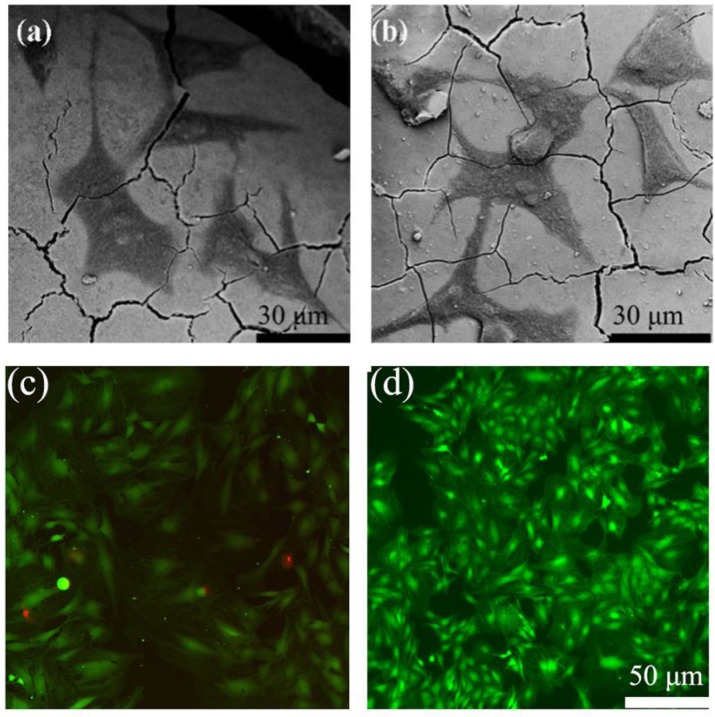
The attachment of hBMSCs on the BMP-2 loaded 1393 scaffolds (a) and 1393@MBG scaffolds (b) after culturing for 2 days; The live (green)/dead (red) staining for the 1393 (c) and 1393@MBG scaffold (d) immersion solution.

**Figure 7:**
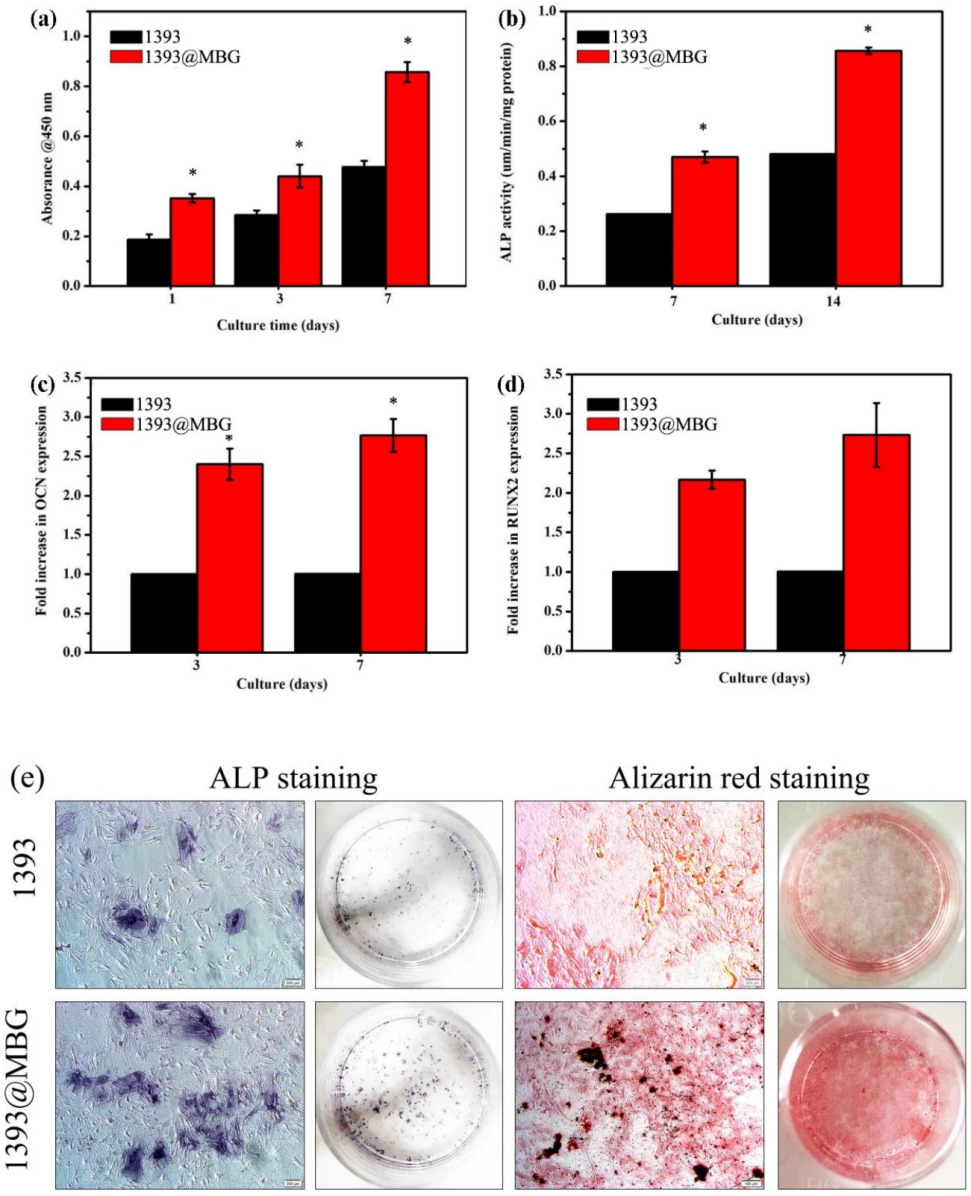
Quantitative measurement of cell proliferation by CCK-8 assays (a) and ALP activity (b) of hBMSCs cultured on the BMP-2 loaded 1393 and 1393@MBG scaffold; The rt-PCR analysis of the osteogenic genes OCN (c) and RUNX2 (d) expressed by hBMSCs cultured on the BMP-2 loaded scaffolds; (e) The ALP staining and ARS staining of hBMSCs cultured with immersion solution of BMP-2 loaded 1393 and 1393@MBG scaffolds for 10 days; mean ± SD; n = 5. *Significant difference when compared to 1393 (*p* < 0.05).

In order to determine the cell response ability of 1393@MBG scaffolds, we loaded BMP-2 on 1393 and 1393@MBG. From the FESEM images, we can easily find that cultured hBMSCs can spread well on the surfaces of 1393 and BMP-2 loaded 1393@MBG group after 48 hours (Fig. 6 a, b). The morphology and prominent filopodia of the hBMSCs were also clearly observed by these images. The viability of the cells in the scaffold were evaluated. As shown in Fig. 6 c, d, cells exhibited green fluorescence, confirming that most cells were alive in the 1393 and 1393@MBG groups. Furthermore, the cell proliferation of hBMSCs increased remarkably in the 1393@MBG groups. Significant growth of cells can be clearly observed in 1393@MBG sample when compared to the 1393 sample on day 3 and 7 (*p* < 0.05). Also, a substantial and significant increase of ALP activity for 1393@MBG group compared to that of 1393 scaffolds was found at 7 and 14 (Fig. 7 b), this might be that the higher BMP-2 loading amount of 1393@MBG group. On the 10^th^ day, ALP staining images further demonstrated that the cells co-cultured with the BMP-2 loaded 1393@MBG group produced more ALP than that of the BMP-2 loaded 1393 group (Fig. 7 e). Meanwhile, macroscopic and microscopic alizarin red staining images revealed that the hBMSCs in the 1393@MBG group generated more ECM mineralization than those in the 1393 group at the 10^th^ day. Therefore, the *in vitro* cell results suggested that BMP-2 loaded 1393@MBG scaffolds exhibited better osteogenic effects than that of the BMP-2 loaded 1393 scaffolds.

As we know, RUNX2 and OCN are two typical osteogenic differentiation markers, herein, we have further evaluated the effects of the BMP-2 loaded 1393@MBG and 1393 scaffold on the differentiation of hBMSCs, and the expressions of RUNX2 and OCN were also determined. The results showed that both RUNX2 and OCN regulated gene expression in the 1393@MBG group was higher than that of the 1393 scaffolds on both day 7 and 14. Together, these data prove that the BMP-2 loaded 1393@MBG scaffolds can significantly better stimulate the proliferation and osteogenic differentiation of hBMSCs compared to the 1393 scaffolds.

### The new bone tissue regeneration of the scaffolds *in vivo*

**Figure 8:**
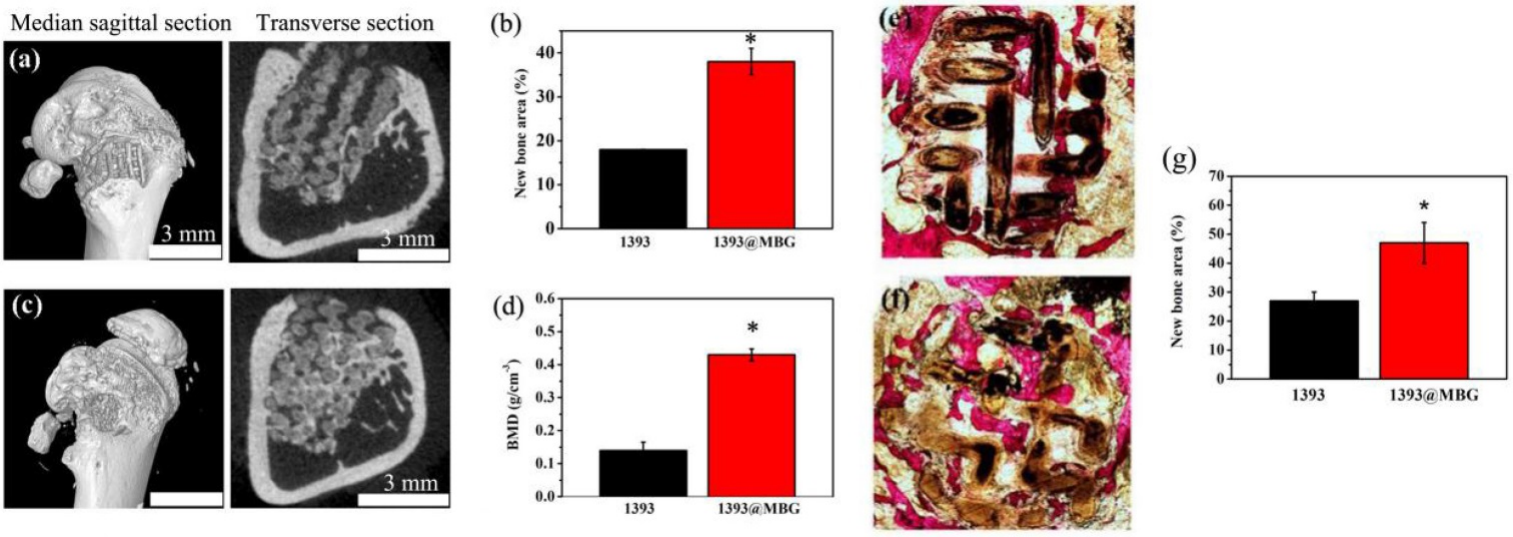
Micro-CT evaluation of bone regeneration in the rat cylindrical defects implanted with the BMP-2 loaded 1393 (a, b) and 1393@MBG (c, d) scaffolds; Transmitted light images of van Gieson picrofuchsin-stained sections of the rat defects implanted with BMP-2 loaded 1393 (e) and 1393@MBG (f) scaffolds; (g) percent new bone area in the defects implanted with the scaffolds and in the unfilled defects; mean ± SD; n = 3. *Significant difference between groups (*p* < 0.05).

A femoral cylindrical defects model was used to evaluate the *in vivo* stimulatory effect of the fabricated scaffolds for bone tissue regeneration. The micro-CT images exhibited a larger quantity of new tissue in the BMP-2 loaded 1393@MBG group than that of 1393 group. From the cross-sectional view, well-integrated tissue and many new tissue was found in the 1393@MBG group, while that of 1393 group appeared to be lower. Moreover, BMD data exhibited that the new bone regeneration of 1393@MBG sample was significantly better than that of the 1393 sample (Fig. 8 a, b, d).

Van Gieson's staining was conducted to further investigate the efficacy of BMP-2 loaded 1393@MBG scaffolds in bone tissue regeneration (Fig. 8 e, f, g). The image showed that void space and fibrous tissue were found in 1393 group, however, well integrated new bone tissue was filled in that of 1393@MBG group. Quantitative analysis also indicated that the 1393@MBG group was significantly better than that of 1393 group. The analysis of both micro-CT and Van Gieson's staining revealed that the BMP-2 loaded 1393@MBG scaffolds had a better osteogenic capacity than that of 1393 scaffolds.

## Discussion

To date, many bone repair materials have been developed for treating bone defects; however, their bone-forming ability cannot meet the demands of patients, especially those with osteoporosis or other orthopedic relative diseases. Some active chemical drugs or cytokines have been employed to repair local bone defects, while the problems related to the activity of these sensitive drugs or cytokines have been seldom focused on. Hence, through this research, novel bone repairing grafts with high feasibility of multi-functionalization or vehicle-like capacity for drug or cytokines delivery were developed for the first time to be used as bone repair implants or bone related diseases.

From the above results we can clearly see that the coating of MBG on 1393 scaffolds is a viable way to endow basic scaffolds with multifunctional properties. Herein, one novel drug delivery scaffold has been built and it can successfully load BMP-2 and DEX to promote bone repair or antibacterial properties *in vitro* and *in vivo*. The basic 3D printing scaffolds had a well ordered and uniformed macropore structure, and the size of the pores were around 300 μm which mimicked the hierarchically 3D pore structures and size of human bone. The well-ordered microporous scaffolds can load high amount of drugs or BMP-2. At the same time, such microporous and interconnected structures are essential for cells to attach, migrate, and transport oxygen, nutrient supply and metabolic waste 35, 36. The bioactivity and osteogenesis changes caused by the MBG coating were evaluated by culturing with hBMSCs *in vitro* and a rat femoral defect model *in vivo*. The DEX loaded 1393@MBG scaffolds exhibited excellent antibacterial properties. The 1393@MBG scaffolds loaded with BMP-2 can better promote proliferation and ALP activity of hBMSCs and upregulate the osteogenesis-related genes expression than the control group.

The drug load mechanism was detailly investigated as follows. Mesoporous silicas exhibited controlled release ability by physical absorption drugs, which will have little effect on the activity of sensitive drugs. As reported, there are two main factors that could affect the drug/matrix physical interaction: the functional groups in drug loading system and the structure of the pore wall 30, 31. It is generally believed that drugs with large molecules will restrict the adsorption and controlled release of materials. In this study, three kind of drugs (DNA, BMP-2 and DEX) were used to evaluate the MBG drug loading ability and the corresponding results indicated that the MBG has better drug-controlled release ability for drugs with smaller molecules. On the other hand, the existence of silanol groups in the MBG channel walls, will lead to the formation of weak inter-bonds with drugs by electric charge absorption, which hold drugs and allow them to be released in a sustained manner. As the MBG wall and pores contain free silanol groups which are positively charged, it can react with some appropriate functional groups such as PO_4_^3-^ and cholesterol group with negative charged molecules of DNA and DEX. Due to the balance of electrostatic interaction, the MBG will absorb DNA and DEX easier than BMP-2 (Fig. 4).

The bioactivity and osteogenesis changes by the MBG coating layer were evaluated by culturing the scaffolds with hBMSCs *in vitro*. The osteogenesis ability of the 1393@MBG scaffolds was determined by CCK-8 assay, ALP activity, ALP staining and mineralization evaluation assays. ALP is one of the key enzymes in BMSC differentiation and osteogenesis, and higher ALP activity indicates an enhancement of bone formation *in vitro*. MBG exhibited a highly mesoporous texture and high surface area which will endow it with a higher surface reaction rate that will result in a faster release of some types of dissolved ions in immersion solution. The high local concentrations of dissolved ions within MBG channels contributed to the quicker deposition of HA 32. Furthermore, a higher degree of surface Si-OH of MBG provided more sites for nucleation on the calcium phosphate layer, which also can lead to higher amounts and faster formation of HA 30. While for the common non-porous material, the 1393 sample, the lower surface area leads to slower reactions and less calcium phosphate nucleation enrichment. Besides, we can clearly see that the coating of MBG on 1393 scaffold can slow down the degradation process, including the pH value changes of scaffold and the dissolved ions release such as Si ions (Fig. 7). The reason for this phenomenon was that the surface of 1393@MBG scaffold will form a loose silicon-rich layer when MBG has degraded, that will further produce HA and prevent ion release and thus led to a slower degradation process. The surface formed silicon-rich layer can absorb cell adhesion-associated proteins 42. In the end, for the 1393 and 1393@MBG group we can clearly see that the release of some types of ions has been slowed down, this can reduce the toxicity generated by some of the released ions. This may explain why the 1393@MBG possessed much better osteogenesis than that of the 1393 group.

Different surface roughness also affects the bioactivity of materials, it has been reported that the rougher it is, the more beneficial it is for cell proliferation 33. The larger the amount of calcium released, the higher the deposition of crystalline apatite, whereas a low-crystallinity Ca-P deposition covered the surface of 1393 sample. This can be seen from the XRD data that showed that the MBG group has better crystalline apatite and bioactivity than that of the 1393 group (Fig. 4). It is well known that the osteoblast-like cells exhibit roughness-dependent phenotypic characteristics 37, 38 and prefer to attach more readily to a rougher microtopography surface. This may be attributed to the increase of free energy on the rougher surface 33, 39. Martin et al. have reported that 1.5 ∼2.0 μm is the optimal value for osteoblast-like cell adhesion 40. Wennerberg have reported that an average surface roughness on the order of 1∼1.5 μm can result in superior bone fixation 41.

Furthermore, to study the mechanism of the scaffolds in osteogenesis, rt-PCR analysis was employed to measure the expression levels of osteogenesis-related genes. The results of the rt-PCR analysis revealed that both the OCN and Runx2 of the 1393@MBG group were significantly upregulated compared to that of 1393 group, which indicated that the 1393@MBG scaffold can promote the differentiation and osteogenesis of hBMSC. Nevertheless, further study is required to elucidate the specific mechanisms.

In addition, an *in vivo* study with rat femoral defects model was conducted to confirm the biofunction of these scaffolds in bone repair. 3D reconstruction of micro-CT images allowed visualization of the effects of BMP-2 loaded 1393@MBG scaffolds, and the results were verified by quantitative evaluation of BMD and BV/TV. Meanwhile, VG staining also showed more bone and collagenous matrix formation at the defect site. Combined with the *in vitro* results, the highly co-expressed osteogenic factors can act synergistically to recruit hBMSCs into the bone defects, which increases cell survival and promotes cell ossification 3, 43, 44, resulting in a better osteogenic capacity of 1393@MBG scaffolds than that of 1393 scaffolds.

The above *in vitro* and *in vivo* results suggested that the novel vehicle-like drug delivery 3D printing 1393@MBG scaffold could be a promising candidate for bone repair, relative bone disease treatment. Despite these advantages, there are disadvantages of the MBG material; the disadvantages are its' brittleness which renders the material with low mechanical strength, the coating layer easily peels off and the material has a high rate of degradation coupled with an unstable surface/interface. Further studies are needed to make breakthroughs in this field.

## Conclusion

In summary, we have successfully fabricated a novel vehicle-like drug delivery scaffold built by 3D printing technology for bone repair. The MBG coating layer has retarded the degradation process and slowed down the release rate of ions. The higher bioactivity of MBG coating endowed the 1393@MBG scaffold with a rougher surface and higher amounts of HA generation. Three kinds of drugs (DNA, BMP-2 and DEX) were applied to evaluate the drug loading and controlled release ability of the as made MBG. The corresponding results indicated that the MBG has better drug controlled release ability for drugs which were smaller molecules and positively charged. Herein, we applied DEX and BMP-2 on the 1393@MBG scaffolds to endow the scaffolds with antibacterial and osteogenesis properties. We have investigated the response to the functional scaffolds of hBMSCs *in vitro* and the osteogenic capacity in rat femoral defects *in vivo*. Results showed that the coating of MBG on 1393 scaffolds were a viable way to enhance the proliferation and ALP activity of hBMSCs and upregulate osteogenesis-related genes expression. The prepared BMP-2 loaded 1393@MBG scaffolds significantly improved bone regeneration in the osseous defects at 12 weeks post-implantation. These results suggested that the novel drug delivery 1393@MBG scaffolds could be a promising candidate for the use in bone tissue repair and relative disease treatment.

## Supplementary Material

Supplementary figures.

## Figures and Tables

**Figure 5:**
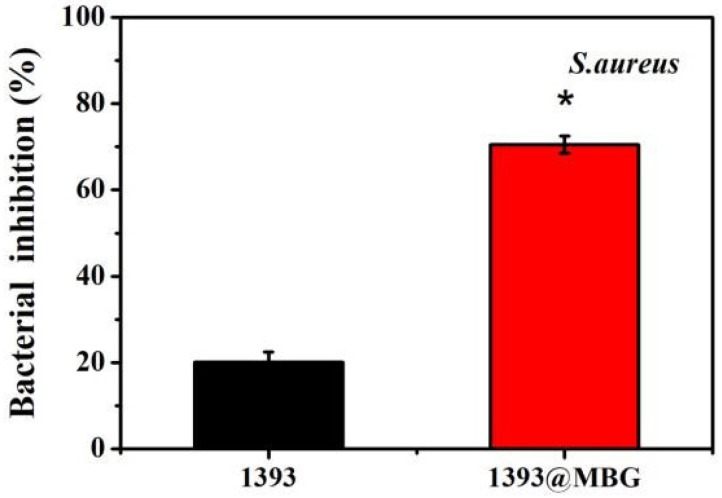
Percent bacterial inhibition *S. aureus* by the scaffolds after incubation for 24 h; mean ± SD, n = 5. *Significant difference when compared to 1393 (*p* < 0.05).
