# A novel vehicle-like drug delivery 3D printing scaffold and its applications for a rat femoral bone repairing *in vitro* and *in vivo:* Erratum

**DOI:** 10.7150/ijbs.59031

**Published:** 2021-02-20

**Authors:** Hui Wang, Zhengwei Deng, Jing Chen, Xin Qi, Libing Pang, Bocai Lin, Yan Teik Yuin Adib, Na Miao, Deping Wang, Yadong Zhang, Jiusheng Li, Xiangqiong Zeng

**Affiliations:** 1 Laboratory for Advance Lubricating Materials, Shanghai Advanced Research Institute, Chinese Academy of Sciences, Shanghai 201210, China.; 2 Department of Orthopedics, Fengxian District Central Hospital Affiliated of Shanghai University of Medicine&Health Sciences, 279 zhouzhu road, Shanghai 220120, People's Republic of China.; 3 School of Materials Science and Engineering, Tongji University, Shanghai 201804, China.; 4 School of Life Science & Chemical Technology, Ngee Ann Polytechnic, Singapore 599489; 5 Graduate School, Shanghai University of Traditional Chinese Medicine, Shanghai 201203, China; 6 Department of Pediatrics, Maternal and Child Health Hospital of Zaozhuang City, Shandong, China.

In our paper 1, the author has made a mistake by selecting wrong SEM panels during Figure 2 (c, d) and Figure 3 (e, g) preparation of 1393@MBG group. Considering the consistency and the accuracy of SEM data, the authors had repeat experiments and carefully re-examined them by SEM for the refabricated 1393(control group) and 1393@MBG scaffolds. The original figures were attached as supplementary figures. The authors apologize for all these errors and state that these corrections do not change the scientific conclusions of the article in any way.

Figure 2, Figure 3 and the legends should be corrected as follows.

## Supplementary Material

Supplementary figures.

## Figures and Tables

**Figure 2:**
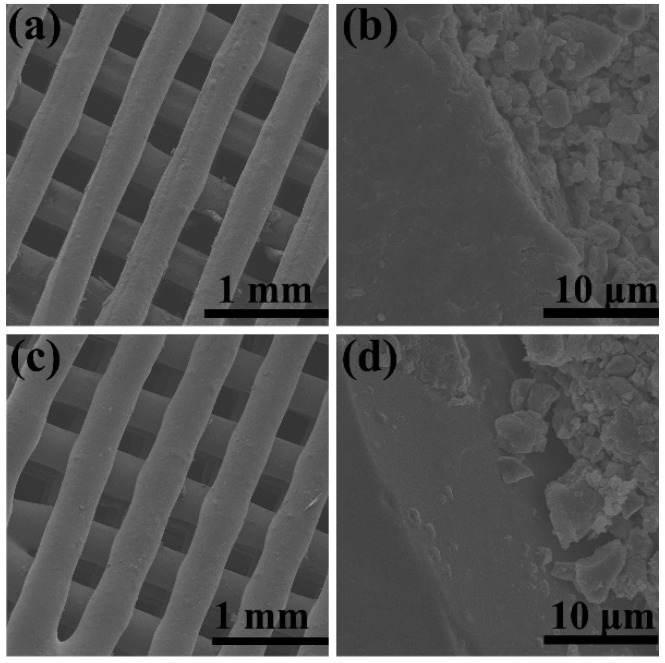
FESEM images of (a, c) as fabricated 1393 and 1393@MBG scaffold; (b, d), the cross section of as fabricated 1393 and 1393@MBG scaffold.

**Figure 3:**
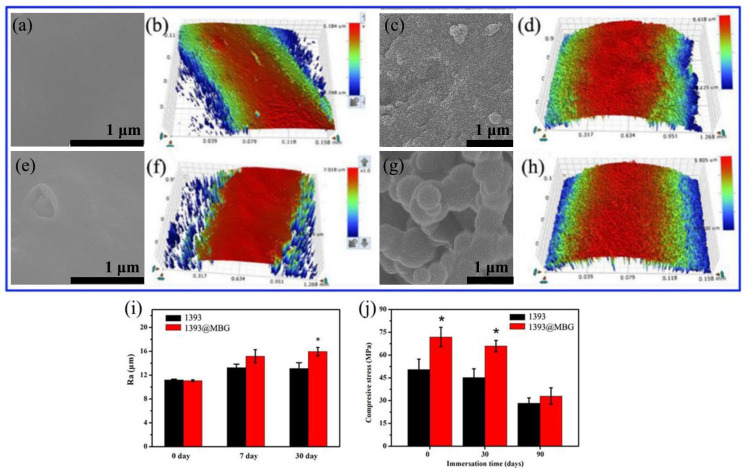
(a, b) FESEM images and the surface profile of as fabricated 1393 scaffold; (c, d) after 30 days immersed scaffold; (e, f) FESEM image and the surface profile of as fabricated 1393@MBG scaffold; (g, h) after 30 days immersed scaffold; (i) The Ra of 1393 and 1393@MBG scaffold surface when immersed from 0 to 30 days; (j) The compressive strength of the 1393 and 1393@MBG scaffold on day 0, 30 and 90. mean ± SD, n = 5. *Significant difference when compared to 1393 (p < 0.05).
