# Supplementary material for: A novel vehicle-like drug delivery 3D printing scaffold and its applications for a rat femoral bone repairing in vitro and in vivo
Source: Int J Biol Sci. 2020 Apr 1;16(11):1821–32. doi: 10.7150/ijbs.37552 (PMC7211168; doi:10.7150/ijbs.37552)
Supplement: Supplementary file 1 — Supplementary figures. [file ijbsv16p1821s1.pdf]

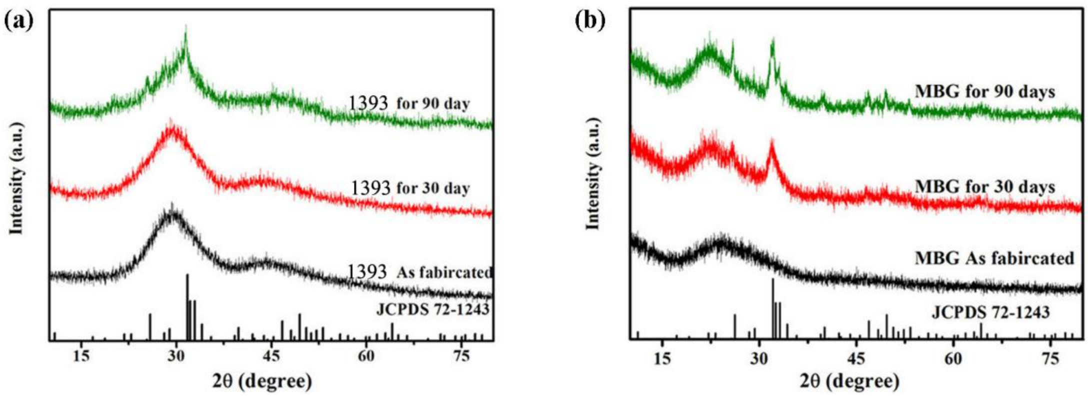

Fig. S1 XRD patterns for 1393 (a) and MBG (b) glass powder as immersed in SBF for 0 and 90 days. A reference hydroxyapatite (JCPDS 72-1243) is shown for comparison.

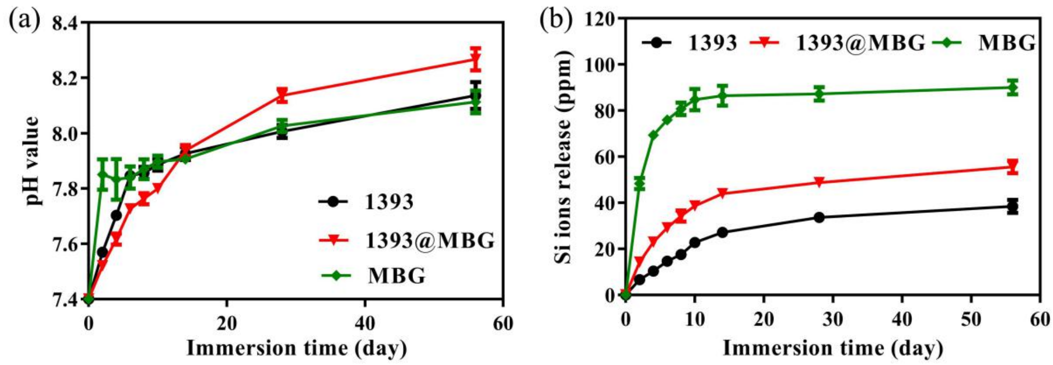

Fig. S2 (a) pH value changes of the immersion medium; (b) Concentration of Si ion released from the scaffold into the medium as immersed in SBF; mean ± SD, n = 5.
