# Supplementary material for: A novel vehicle-like drug delivery 3D printing scaffold and its applications for a rat femoral bone repairing in vitro and in vivo: Erratum
Source: Int J Biol Sci. 2021 Feb 20;17(3):913–4. doi: 10.7150/ijbs.59031 (PMC7975699; doi:10.7150/ijbs.59031)

Control group：

Scaffold

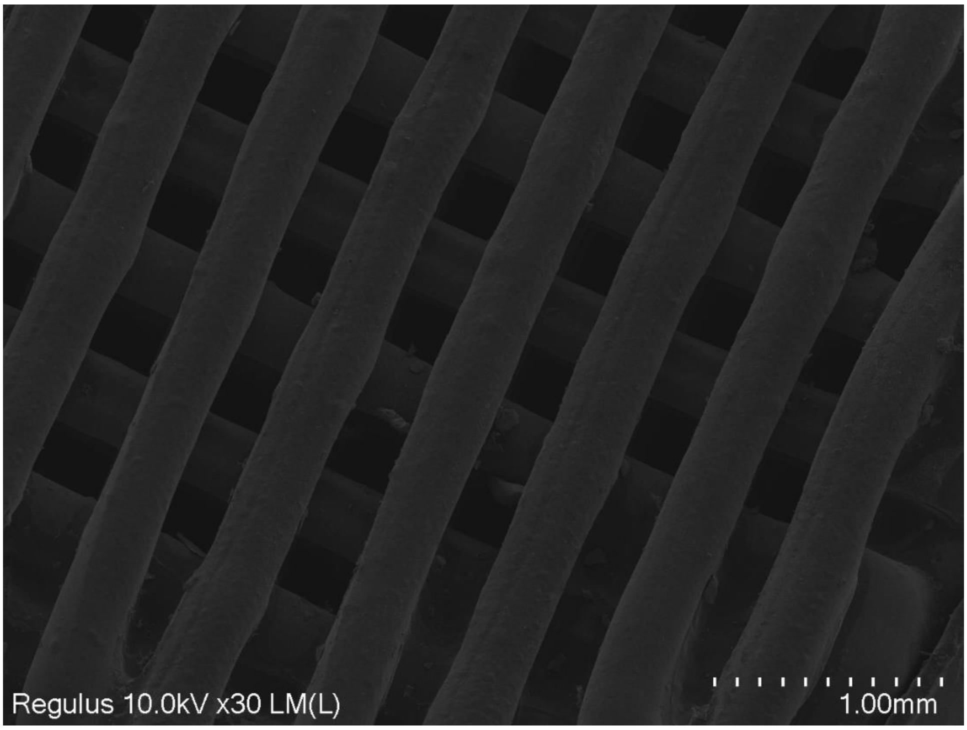

Film

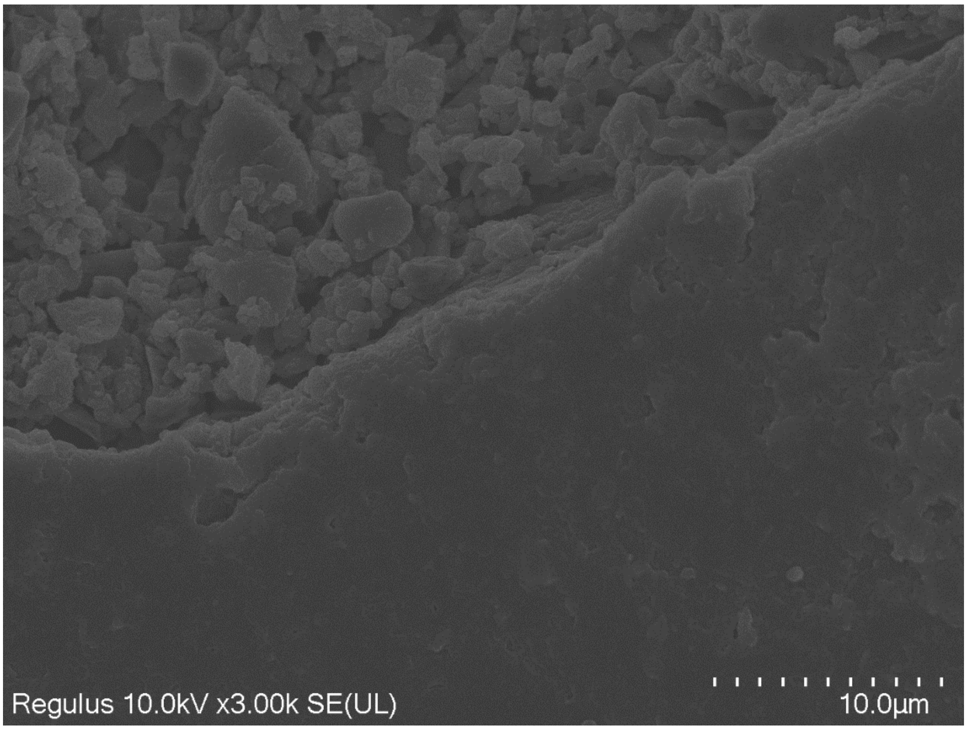

Asfabicated

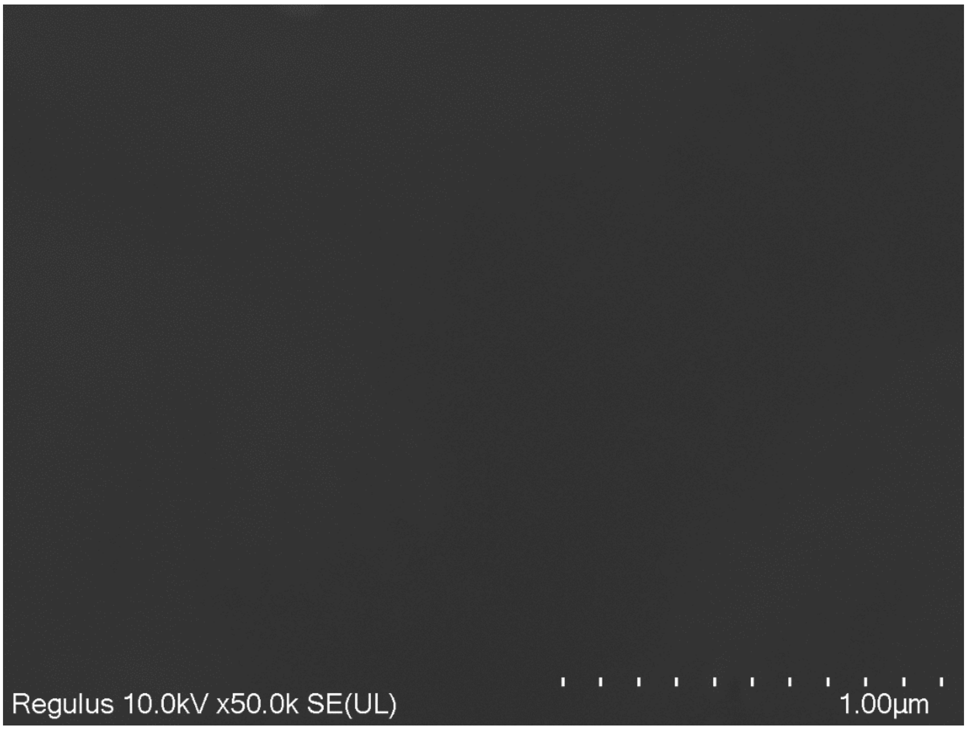

Immersed 30 days

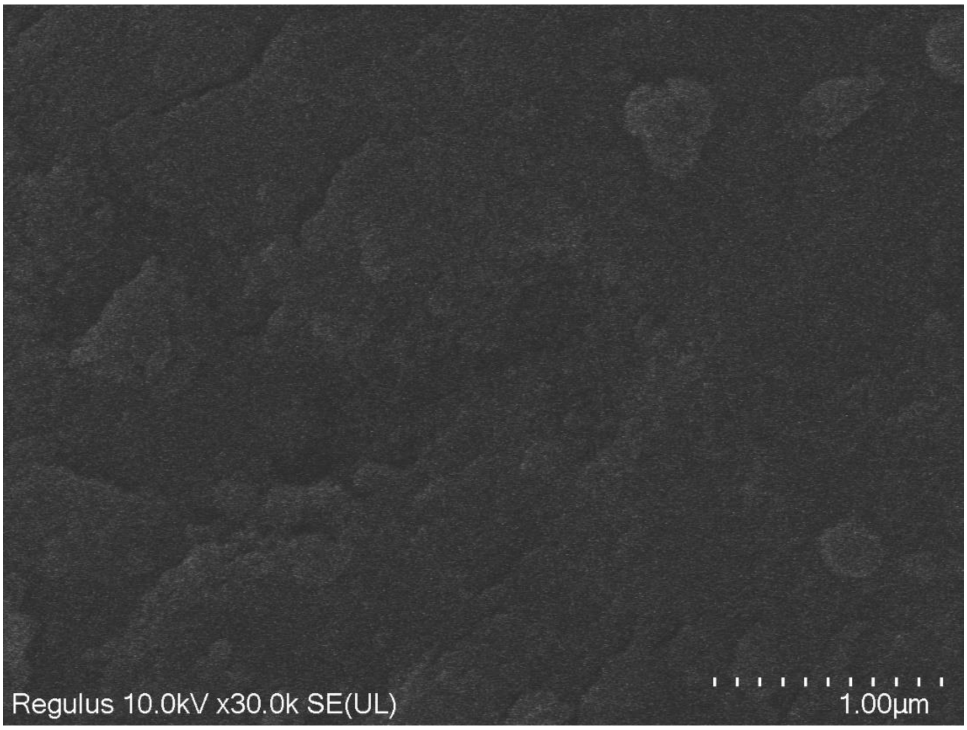

1393@MBG group:

Scaffold

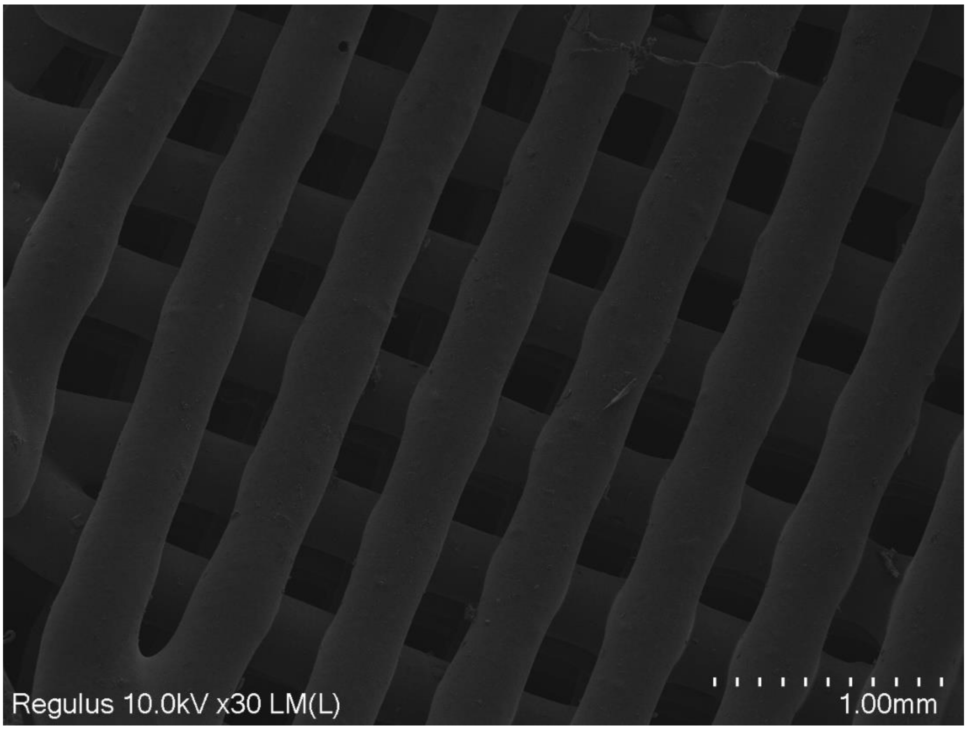

Film

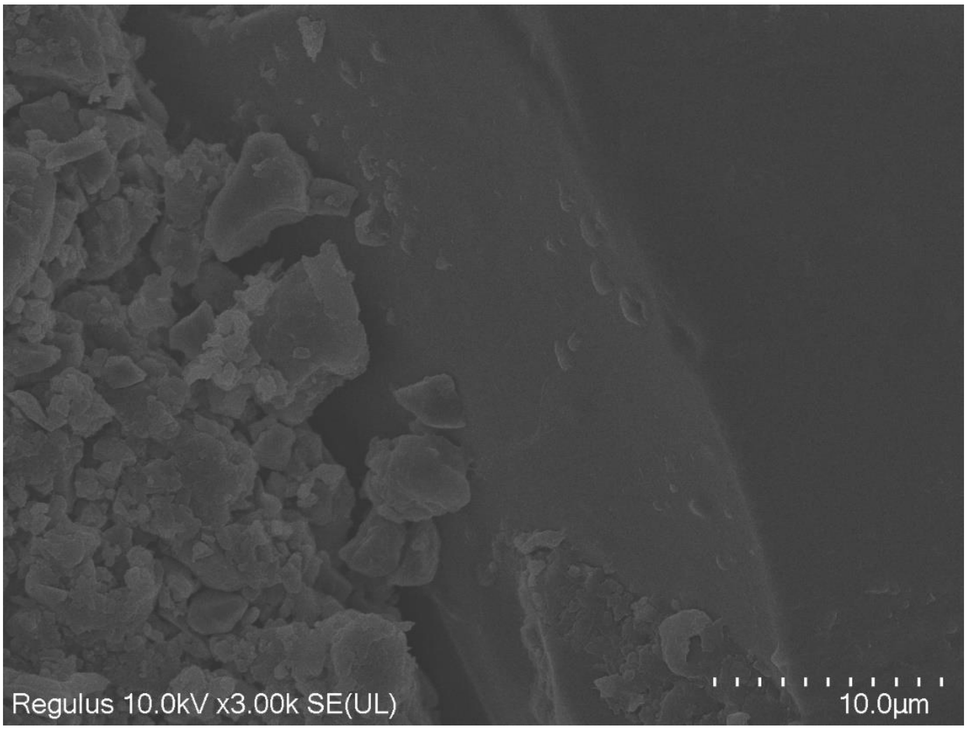

Asfabicated

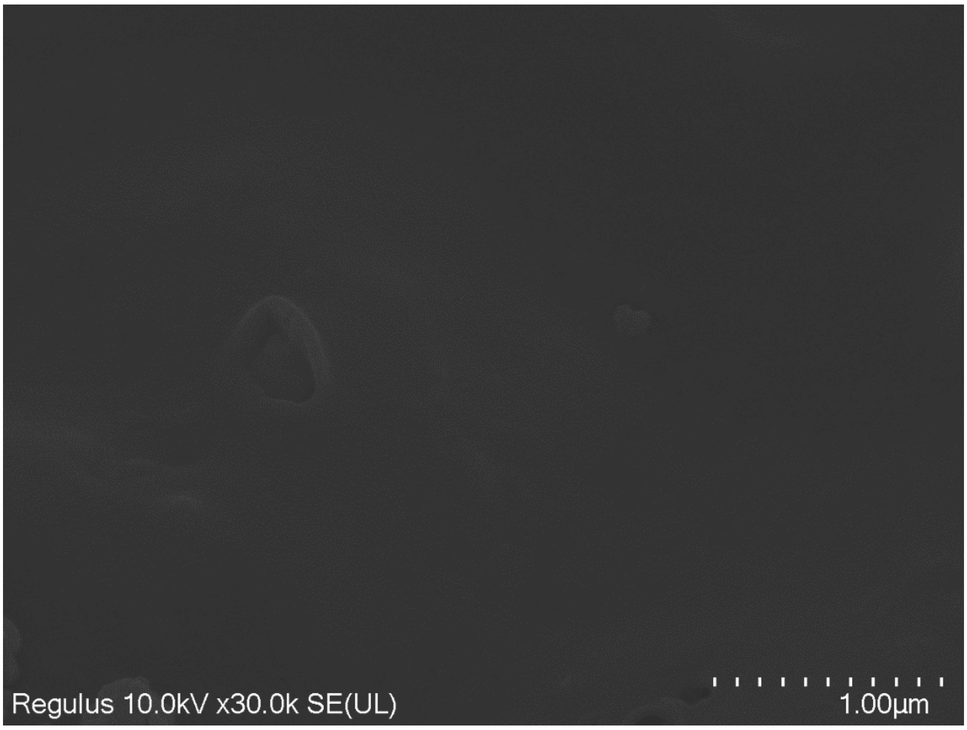

Immersed 30 days

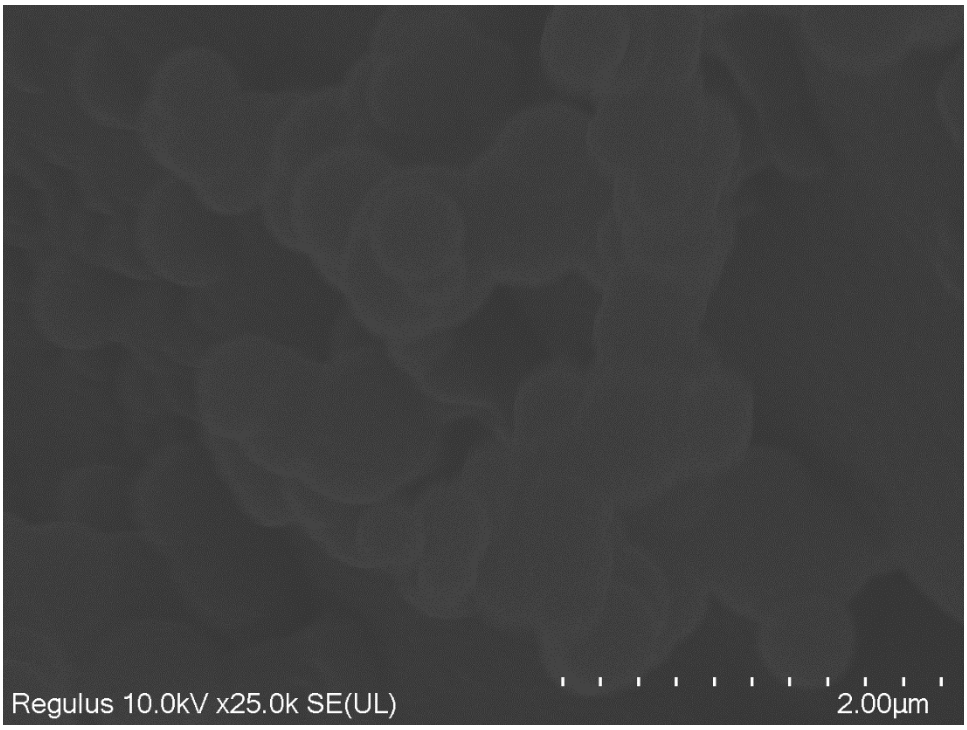

Supplement: Supplementary file 1 — Supplementary figures. [file ijbsv17p0913s1.pdf]
